# Association of bilateral acromiohumeral distance or acromion-greater tuberosity ratio with glenohumeral subluxation for stroke patients with hemiplegia based on ultrasound and radiographic techniques

**DOI:** 10.3389/fneur.2025.1665241

**Published:** 2025-10-03

**Authors:** Hai Yuan, Yan Zhang, Ting Wang, Mao Sheng, Luping Chen, Lili Shen, Pingping Ge

**Affiliations:** ^1^Department of Rehabilitation Medicine, The Second People’s Hospital of Hefei City, Hefei, China; ^2^Department of Rehabilitation Medicine, The Affiliated Hefei Hospital of Anhui Medical University, Hefei, China; ^3^Department of Rehabilitation Medicine, The Second People's Hospital of Hefei Affiliated to Bengbu Medical University, Hefei, China; ^4^Department of Radiology, The Second People’s Hospital of Hefei City, Hefei, China; ^5^Department of Ultrasound, The Second People’s Hospital of Hefei City, Hefei, China

**Keywords:** stroke, hemiplegia, glenohumeral subluxation, radiography, ultrasonography

## Abstract

**Introduction:**

Glenohumeral subluxation (GHS) is notably a prevalent musculoskeletal issue among individuals experiencing hemiplegia following a stroke. This research seeks to assess the association between the ratio of bilateral acromionhumeral distance (AHD) or acromion-greater tuberosity (AGT) obtained via ultrasound or radiographic techniques and the severity of GHS.

**Methods:**

Data were collected from bilateral measurements using ultrasonography or radiography for healthy participants based on the informed consent of each participant, and the ratio of AHDs or AGTs between the left and right sides was computed. Two measurements were employed to ascertain the reliability of the ratio. The relation of the ratio with the severity of GHS was explored based on the correlation analysis for stroke patients with hemiplegia.

**Results:**

A total of 22 healthy participants were assessed, revealing an intra-class correlation coefficient (ICC) of 0.90 (*p* < 0.05) for the AGT ratio. Similarly, the ICC for the AHD ratio with 28 healthy participants, determined through radiographic evaluation, yielded an identical result of 0.93 (*p* < 0.05). In addition, no statistical differences in characteristics between ultrasonography and radiography groups. 47 cases of stroke patients with hemiplegia were included, and GHS was clearly diagnosed. A statistical correlation was taken between the acromio-humeral interval and the ratios in patients with stroke hemiplegia (*N* = 21, r = 0.56, *p* < 0.05 for ultrasonography and *N* = 45, r = 0.49, *p* < 0.05 for radiography).

**Discussion:**

The ratio of bilateral AHDs or AGTs, assessed through ultrasound or radiographic method, serves as an important metric for GHS among individuals experiencing hemiplegia following a stroke, thereby facilitating focused rehabilitation strategies.

## 1 Introduction

Stroke poses a considerable challenge to global healthcare due to its severity and the disabling effects it entails (1). Individuals with stroke experiencing significant ongoing weakness in their upper limbs are at an elevated risk of secondary complications, including glenohumeral subluxation (GHS) and shoulder pain (2). The frequency of GHS, as well as shoulder subluxation, a critical outcome of motor dysfunction, shows considerable variability among stroke survivors, ranging from 17 to 81% (3). GHS could restrict upper limb functionality and daily activities of stroke patients, such as eating, dressing, brushing teeth, etc. (4). In addition, GHS may be regarded as a potential contributor to the onset of shoulder pain (5). The alterations in the position of the humeral head leave surrounding soft tissues susceptible to injury, which may subsequently lead to shoulder pain (6). Magnetic resonance imaging assessment revealed bicipital tendon-glenoid labrum injury within the GHS cohort and subluxation may demonstrate increased vulnerability to specific injuries (7).

The objective and quantitative evaluation of GHS can assess the efficacy of various treatment methods. Fingerbreadth palpation is employed to assess subluxation, wherein the evaluator attempting to identify the distance between the acromion and the humeral head (8). However, one limitation of this method is its inability to provide an objective measurement based on variations in fingerbreadth (9). Currently, there is no standardized protocol for fingerbreadth palpation, which results in inconsistent outcomes conducted by different physicians. Consequently, ultrasound and radiographic techniques are increasingly adopted by clinicians to evaluate GHS due to their quantitative characteristics.

The acromion-humeral distance (AHD) measured through radiography acts as an indicator for shoulder subluxation, where an increase in AHD suggests shoulder subluxation due to a stroke. Clinically, subluxation can be quantified by obtaining a radiographic image of the shoulder (10). Nonetheless, the influence of measurement distant on the outcome might be considered (11).

The research has underscored the importance of diagnostic ultrasound in assessing the acromion-greater tuberosity (AGT), which is characterized as the minimum distance from the lateral edge of the acromion to the humeral head (12). Multiple studies have investigated the intrarater reliability of AGT measurements in healthy individuals (13) as well as in patient groups (14). The ultrasound could be considered as repeatable as radiography when specific standardized measures are applied and followed (15). Nonetheless, the AGT measurement obtained through ultrasound is considerably affected by individual variations, such as supraspinatus tendon thickness (16), making it challenging to establish a standardized approach for assessing GHS.

Therefore, establishing a standardized method for evaluating GHS that is not influenced by the aforementioned conditions is of great significance. The utilization of the ratio may address the above challenges associated with the direct measurement of GHS values. This study first investigates the reliability of the ratio of bilateral distances (AHD or AGT) in healthy individuals via the radiography or ultrasonography. It subsequently examines stroke patients with GHS resulting from hemiplegia to further explore the validity of the ratio. The ultimate goal of our study is to develop a valuable evaluation standard that can guide the rehabilitation treatment of GHS in clinical settings.

## 2 Methods and methods

### 2.1 Healthy participants

**Figure 1:**
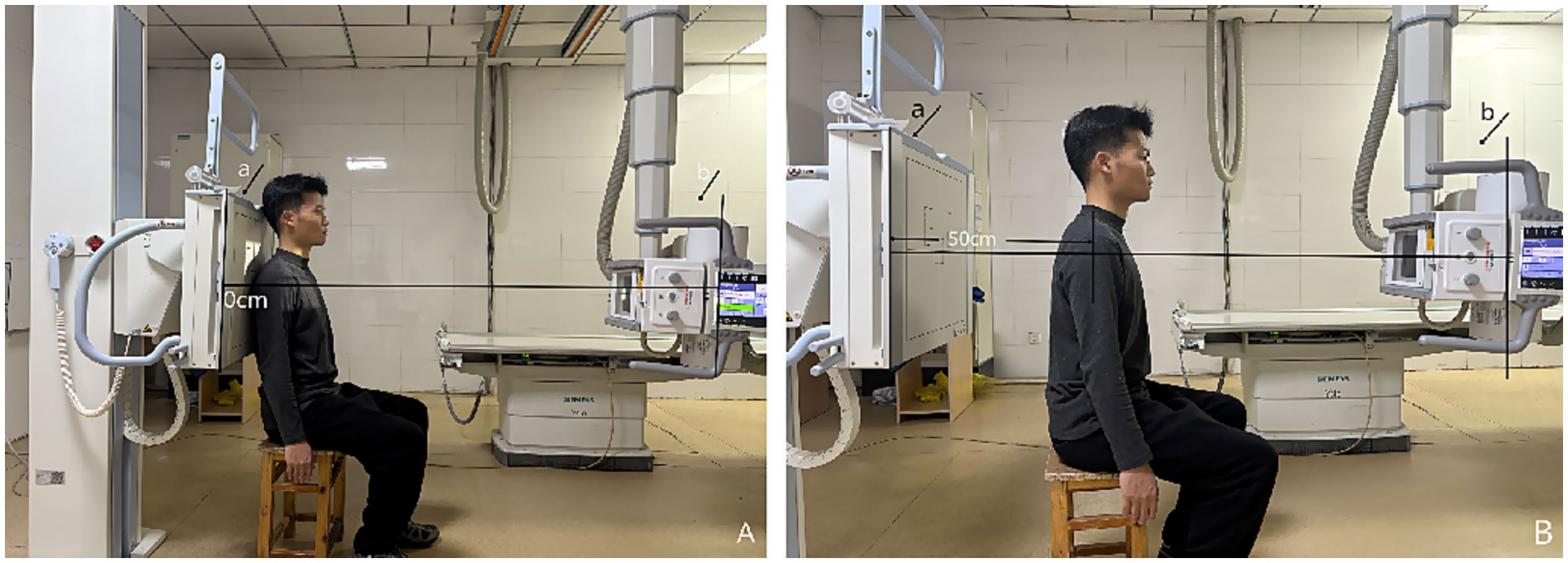
Standard position for radiography of the shoulder joint. **(A)** The distance from individuals to the flat panel detector is 0 cm. **(B)** The distance from individuals to the flat panel detector is 50 cm. a—The flat panel detector, b—The tube.

**Figure 2:**
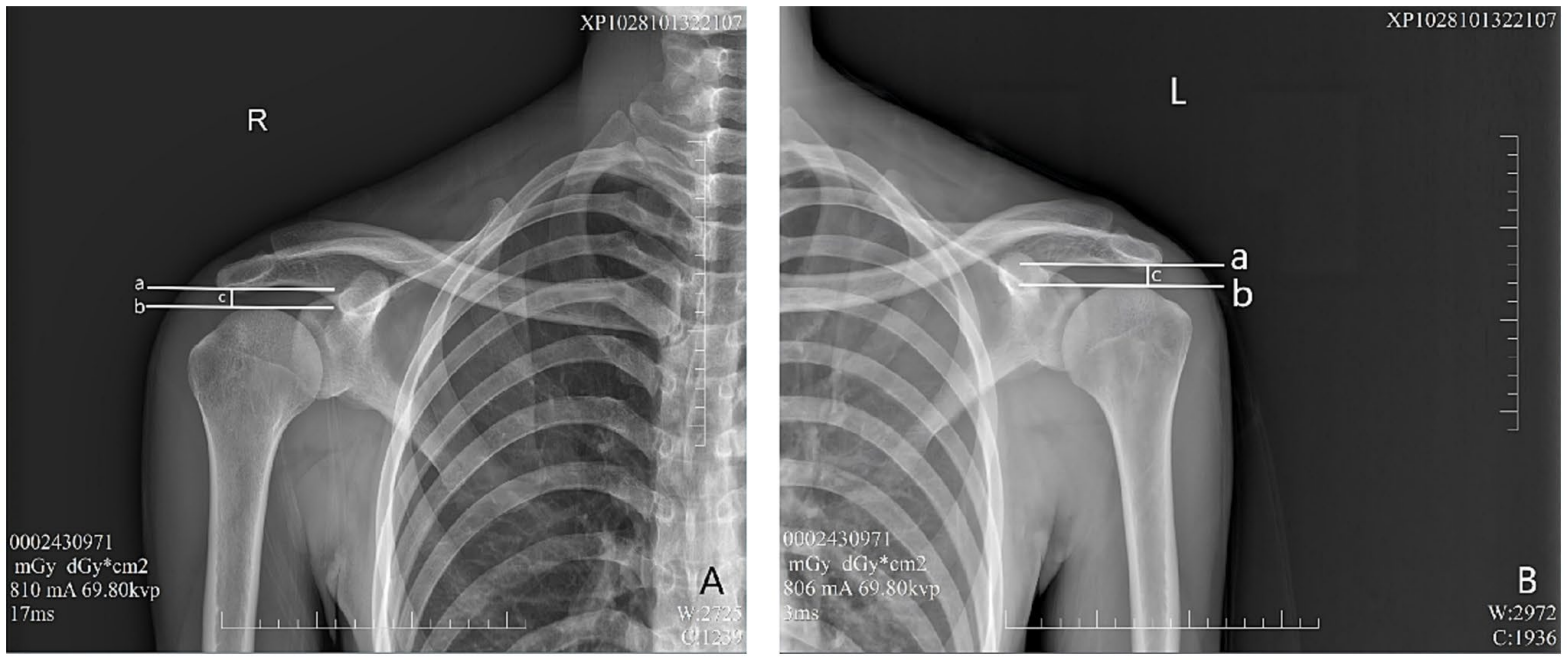
Evaluation of AHD distance from the healthy participant to the flat plane detector at 0 cm. a—A horizontal vertical line across the lowest point of the acromion. b—A horizontal line across the top of the humeral head. c—Distance between lines **A** and **B**, as well as AHD. **(A)** Right shoulder, **(B)** Left shoulder.

**Figure 3:**
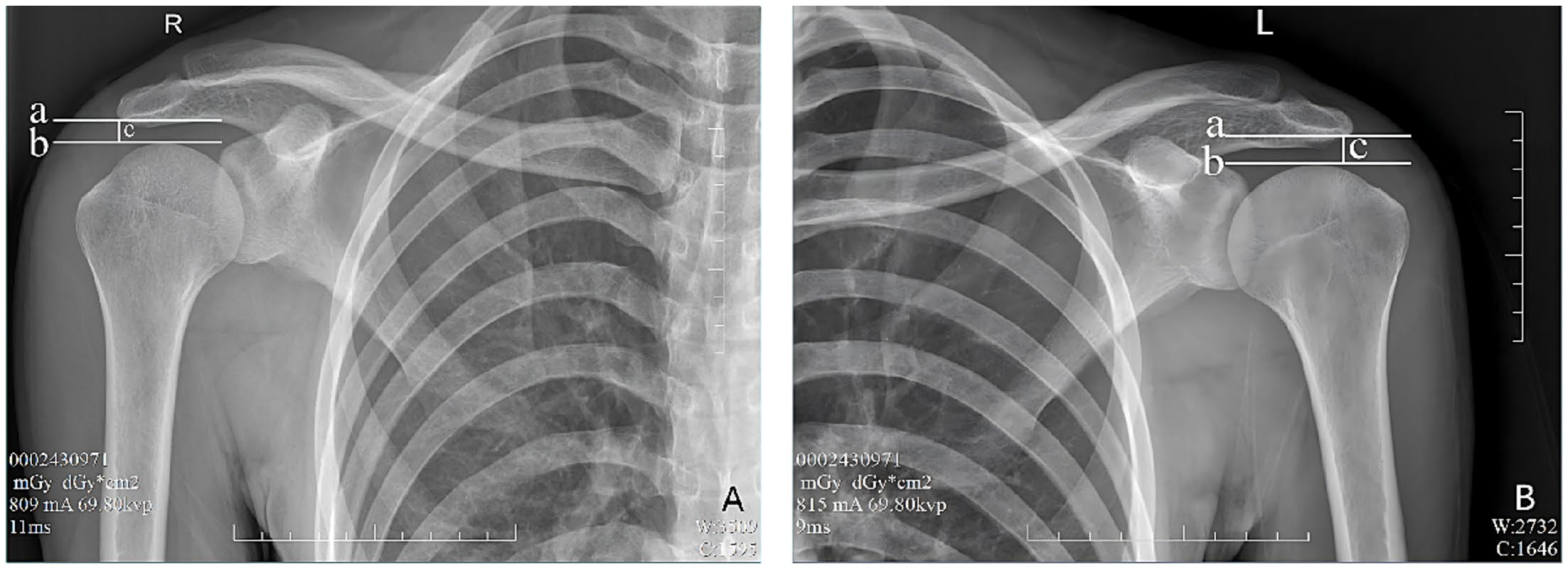
Evaluation of AHD distance from the healthy participant to the flat plane detector at 50 cm. a—A horizontal vertical line across the lowest point of the acromion. b—A horizontal line across the top of the humeral head. c—Distance between lines **A** and **B**, as well as AHD. **(A)** Right shoulder, **(B)** Left shoulder.

In the evaluation of AHD during radiographic procedures, the research included 28 healthy participants based on the informed consent of each participant. During the assessment via radiography (Simens Yiso, Fluorospot compact imaging systems, Germany) (Parameter settings: tube voltage 70 kV, automatic tube current, and small focal spot), participants were asked to maintain an upright position with their arms resting comfortably alongside their bodies and palms oriented towards their torso (Figures 1A,B). A vertical reference line was marked from the lowest point of the acromion, perpendicular to a horizontal line drawn from the top of the humeral head, which allowed for the measurement of the distance between the acromion and the humeral head (17) (Winning Health TView 6.1.0, Winning Health Technology Group Co., Ltd.) (Figures 2, 3). And the ratio of the bilateral distances was calculated (Left/Right). These procedures were performed by a single radiologist. It was made that the consistency of the results was evaluated for the distances from the healthy participant to the flat plane detector, measuring at 0 cm and 50 cm (Figures 1–3).

**Figure 4:**
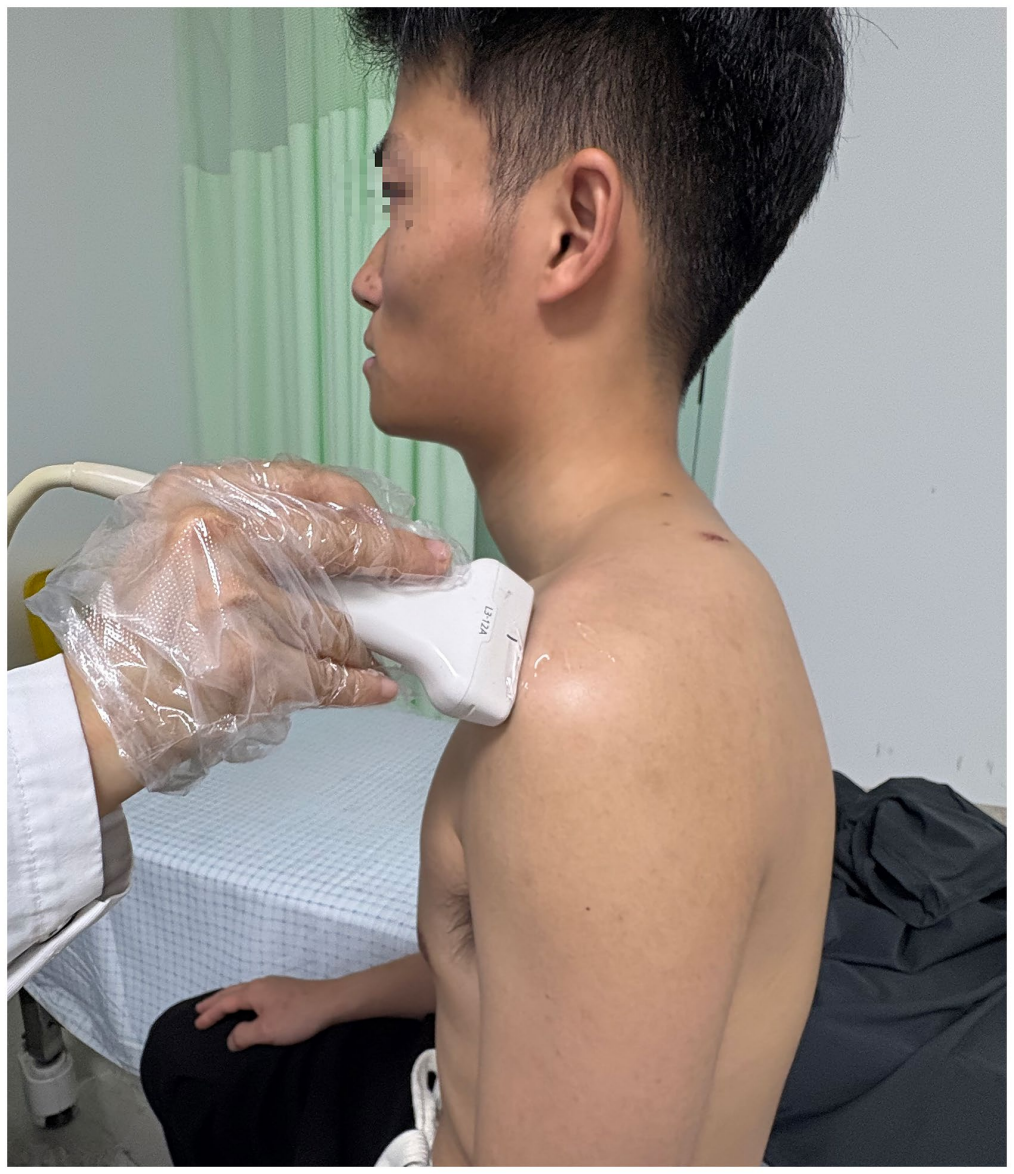
Participants’ standardized position for ultrasound assessment.

**Figure 5:**
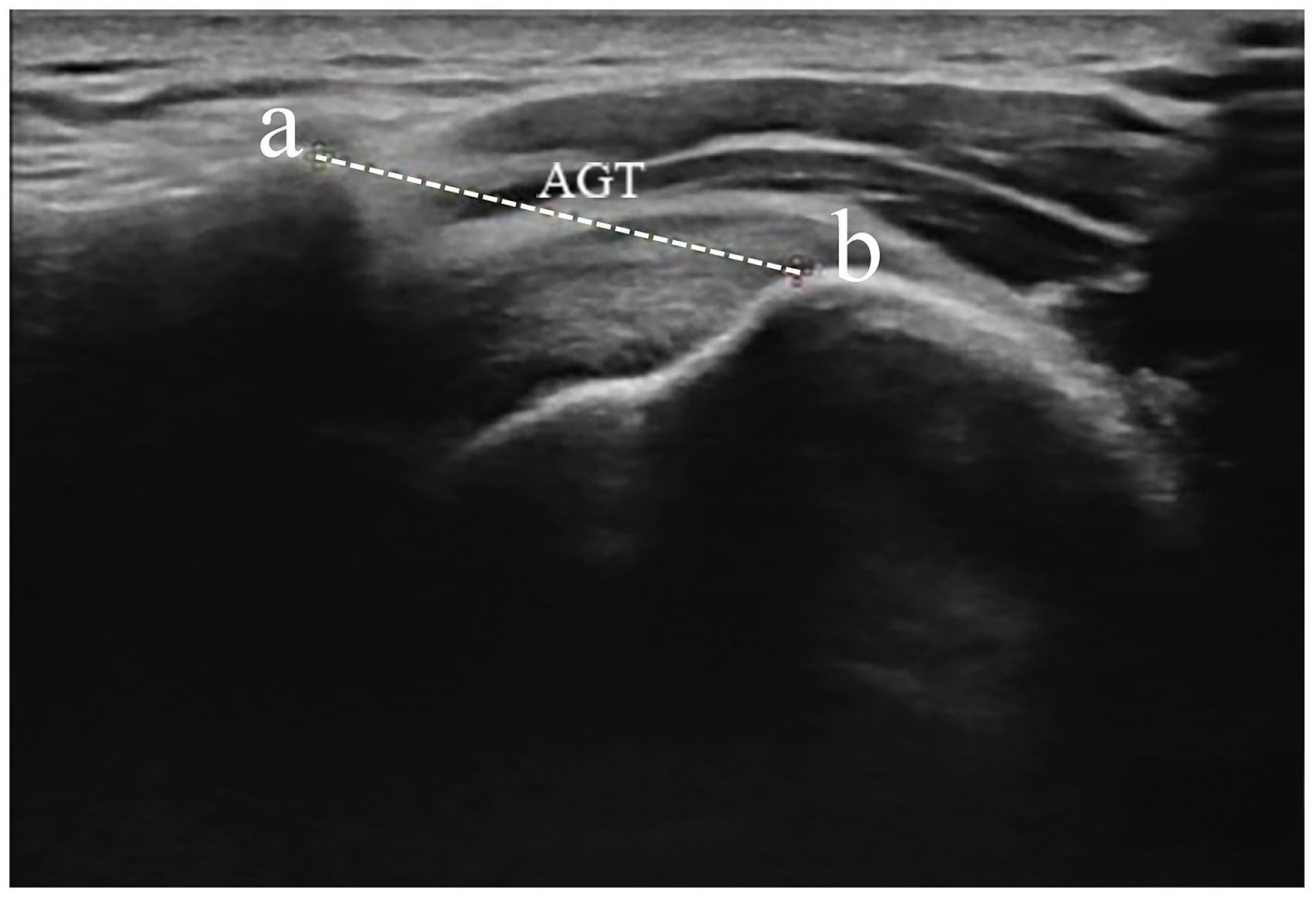
Measurement of AGT distance from the lateral border of the acromion and the nearest superior margin of the greater tuberosity. Dotted caliper represents AGT distance. AGT acromion–greater tuberosity. a—acromion, b—greater tuberosity.

In the evaluation of AGT during ultrasound procedures (RS80A, Samsung, Korea), the research included 22 healthy participants based on the informed consent of each participant. The participants’ posture required is consistent with the posture of radiographic measurement of AHD. The transducer was positioned at the anterior edge of the shoulder within the coronal plane (Figure 4). After the acromion and greater tuberosity of the humerus appeared on the screen, the image was captured, allowing for the measurement of the shortest distance between the acromion and the greater tuberosity (18) (Figure 5). Data from bilateral AGTs were documented, and the ratio of the bilateral distances was calculated (Left/Right). Two separate measurements were utilized to evaluate reliability. These procedures were performed by a single ultrasonologist.

In addition, to investigate whether the outcomes from radiography are influenced by the distance of healthy participants from the flat panel detector, we compared bilateral AHDs at two distinct distances: 0 cm and 50 cm, respectively (Figures 1–3).

### 2.2 Stroke patients

#### 2.2.1 Inclusion or exclusion criteria

The inclusion criteria encompassed a confirmed stroke diagnosis, hemiplegia, the ability to sit independently, and a diagnosis of GHS (17). Exclusion criteria comprised pre-existing shoulder ailments that restricted the mobility of the shoulder joint, significant cognitive deficits, inadequate trunk control that compromised the ability to maintain an standard position for GHS evaluation, and fractures surrounding the shoulder joint.

At last, this study involved 47 patients who had experienced a stroke and exhibited symptoms of hemiplegia and GHS. Participants received physical examinations, ultrasound imaging, or radiographic assessment. Approval for this research was obtained from the Ethics Committee of the Second People’s Hospital in Hefei City (2024-Technology-016), and the investigation was conducted in line with the Declaration of Helsinki. The study’s purpose and details were clearly conveyed, and written informed consent was obtained from the individuals for the publication of any potentially identifiable images or data included in this article.

### 2.3 Statistical analysis

Continuous variables were expressed as means with standard deviations (SD). Data regarding GHS parameters were collected from the clinical assessments. Paired t-tests (Student’s t-tests) were utilized to identify significant differences between the AHD or AGT of the affected and unaffected shoulders, as determined by either radiography or ultrasonography. The two-sided AHD or AGT ratio’s reliability was evaluated using the within-group correlation coefficient (intraclass correlation coefficient, ICC). The correlation analysis (the Pearson correlation) was made to explore the validity of this method between the ratio and acromio-humeral distance via radiography. The statistical analysis was performed using SPSS version 27.0, with *p*-values under 0.05 deemed statistically significant.

## 3 Results

### 3.1 Radiographic assessment

Twenty eight participants were evaluated (Table 1). The ICC for the AHD ratio in healthy subjects ranged from 0 cm to 50 cm, consistently recording a value of 0.93 with significance at *p* < 0.05 (Table 1).

**Table 1:** Characteristics of involving healthy individuals (mean ± standard deviation).

Group	N	Age (years)	Weight (kg)	Height (cm)	M: F	ICC
Radiography	28	24.86 ± 5.19	64.89 ± 10.83	170.14 ± 7.50	20:8	0.93^※^
Ultrasound	22	24.41 ± 4.69	69.68 ± 17.65	171.64 ± 8.21	12:10	0.90^※^
Statistic analysis	50	t = −0.32, *p* = 0.75	t = 1.18, *p* = 0.20	t = −0.19, *p* = 0.85	χ^2^ = 1.52, *p* = 0.22	

M, Male; F, Female; ICC, Intra-class correlation coefficient. ^※^*p* < 0.05.

### 3.2 The bias in radiographic measurement

The comparison results of bilateral AHDs at varying distances reveal statistical differences and this source of bias may be present in radiographic measurement (Left: t = −10.72, *p* < 0.05; Right: t = −10.21, *p* < 0.05) (Table 2).

**Table 2:** The differences of bilateral AHDs between 0 cm and 50 cm (mean ± standard deviation).

Group	Left AHD	Right AHD
0 cm	8.10 ± 1.74	7.99 ± 1.69
50 cm	10.51 ± 1.93	10.43 ± 2.12
Statistic analysis	t = −10.72, *p* < 0.05	t = −10.21, *p* < 0.05

### 3.3 Ultrasound assessment

Twenty two participants were included (Table 1). In a similar manner, we utilized the ICC to evaluate the reliability of the AGT ratio. The ICC for the AGT ratio was found to be 0.90 (*p* < 0.05) (Table 1).

In addition, no statistical differences in characteristics between radiographic and ultrasound assessments (Table 1).

### 3.4 Clinical evaluation

A total of 47 patients (average age 56.60 years, standard deviation 11.86)—comprising 16 with ischaemic strokes and 31 with haemorrhagic strokes—were admitted to the rehabilitation unit, which included 28 males and 19 females for this research. Evaluations took place with an duration from 12 to 301 days after the stroke onset. Among these subjects, two were identified as Brunnstrom stage I, 15 as stage II, 25 as stage III, and four as stage IV, and one as stage V (Table 3). Notably, the measurements for the affected shoulder were significantly higher than for the unaffected side in both radiographic (N = 45, 21.07 ± 7.13 vs 10.10 ± 3.36, t = 10.10, *p* < 0.05) and ultrasound (*N* = 21, 23.38 ± 2.54 vs 20.48 ± 1.17, t = 6.43, *p* < 0.05) assessments of GHS. In addition, a significant relationship was identified between the acromio-humeral distances and these ratios, with results showing r = 0.49, *p* = 0.001 for radiography, and r = 0.56, *p* = 0.007 for ultrasonography, both indicating statistical significance (*p* < 0.05) (Table 3).

**Table 3:** Characteristics of involving stroke patients with glenohumeral subluxation (mean ±standard deviation).

Group	N	Age (years)	Ischaemia: Hemorrhage	M: F	L: R	r
Radiography	45	56.02 ± 11.62	16:29	27:18	22:23	0.49^※^
Ultrasonography	21	55.19 ± 11.61	10:11	13:8	7:14	0.56^※^

M, Male; F, Female; L, Left hemiplegia; R, Right hemiplegia. ^※^*p* < 0.05.

## 4 Discussion

Our current research, based on healthy participants, reveals that the radiographic technique exhibits a high level of reliability in the AHD ratio. Similarly, a high ICC for the AGT ratio based on ultrasound was also found, demonstrating strong test–retest reliability. In stroke patients with GHS, an association was observed between the ratio of bilateral AHDs or AGTs, as measured by ultrasound or radiographic technique, and the severity of GHS. It provides a novel method or concept for the clinical evaluation of GHS in stroke patients with hemiplegia. The occurrence of GHS poses considerable obstacles to rehabilitation of the upper limb, including compromised shoulder function, extended durations of hospitalization, and increased depression due to enhanced disability (19, 20). Among the contributors to GHS, the primary factor is the denervation of shoulder muscles linked to brain injuries, specifically the supraspinatus and posterior deltoid, which causes the humeral head to be directed downwards out of the glenoid fossa under the influence of gravity (21). The mass of the upper limb places tension on the joint capsule, along with muscles, tendons, and ligaments, which further exacerbates shoulder subluxation (22, 23). GHS predominantly occurs during the flaccid stage following a stroke (24).

At present, the management of GHS in individuals who have suffered a stroke involves multiple strategies, such as shoulder taping (25), functional electrical stimulation (26). Nonetheless, the effectiveness of these interventions is mainly determined via radiography or ultrasonography, which analyze the AHD or AGT as a result of treatments. This evaluation is notably affected by variations among individuals or the measurement distance. Recently, the clinical assessment techniques comprise the finger breadth (palpation) method (27), radiography (28) and ultrasonography (19).

The radiographic measurement of GHS is taken from the inferior part of the acromion to the superior part of the humeral head, and the difference between the affected and unaffected shoulders is utilized for diagnosed subluxation (29). It has been demonstrated the correlation between radiographic measurement and fingerbreadth palpation (30). Several investigations have utilized radiography to assess the alterations in AHD following the implementation of supportive devices (19, 31). Fujimura et al. (32) performed an anteroposterior radiograph of both shoulders under a stress test to identify subluxation. Alongside explanations regarding the technique for radiography the hemiplegic shoulder, various methods have been proposed to assess subluxation based on the resulting images (16, 33). Nonetheless, these researches address the challenges linked to individual constitutional variations and the influence of both distant and proximal flat plane detector on measurement outcomes when assessing GHS (11, 16). The substantial discrepancies in AHDs based on different measurement distances were found, suggesting that the distance of the flat plane detector can introduce variability in results, ultimately affecting the reliability of these measurements. At last, this variability is exacerbated by physiological differences among individuals, such as variations in body composition (e.g., fat versus thin individuals), which may lead to measurements that fail to accurately represent true values.

We utilize the AHD ratio to mitigate the impact of physiological variations. Our research reveals that participants show a high level of consistency when assessed in various positions of the flat plane detector, suggesting that the ratio has a strong reliability. In addition, the ratio tends to approximate 1, indicating that healthy individuals likely possess nearly identical bilateral AHDs. For patients, this ratio displays a significant correlation with GHS value to exhibit considerable reliability and validity to be possible to objectively and quantitatively evaluate GHS in patients with stroke.

In our research, the distance measured using the ultrasound technique is affected by individual variations. Specifically, a healthy assessment showed a measurement span of 18 mm to 26.9 mm, indicating that these absolute values might not truly reflect the AGT level. Nevertheless, the study demonstrated that the distances between the AGTs of both shoulders are roughly equivalent, indicating a degree of consistency. As a result, the ratio of these distances serves as a more accurate indicator of the reliability of these measurements. Furthermore, this ratio reveals a significant correlation with GHS value in patients, suggesting that the outcomes possess substantial reliability and validity, allowing for an objective and quantitative assessment of GHS in stroke patients.

In recent years, the evaluation of the degree of GHS has increasingly involved ultrasound imaging. The ultrasound technique may offer a benefit compared to fingerbreadth palpation in recognizing patients with minor subluxation (34). Measuring the distance from the AGT can help ascertain the presence of subluxation in patients with hemiplegia (35). One study found outstanding intra-rater (test–retest) reliability for AGT distance measurements recorded by both novice and experienced raters in individuals with post-stroke hemiplegia (36). These findings are consistent with earlier studies focusing on stroke patients (34, 37). It has been noted that dislocation assessments might indicate subluxation if there is a 0.2 cm or greater difference between the affected and unaffected sides (34).

Unexpected results offer additional support for the clinical relevance of ultrasonographic measurement of the AGT, which might be used in diagnosing supraspinatus impingement syndrome (SIS) (38). Rotator cuff tears are one of the underlying causes contributing to the onset of SIS. Cholewinski et al. (39) discovered a notable statistical difference in AGT measurements between the shoulders affected and unaffected by rotator cuff issues. Individuals with rotator cuff tears showed a reduction in the average AGT, with those suffering from more severe rotator cuff tears exhibiting a more significant decrease (29). In light of these results, the disparity in AGT measurements between the unaffected and affected shoulders could function as a diagnostic indicator for recognizing certain shoulder-related disorders. These results highlight the promising role of quantitative shoulder ultrasound in facilitating both research and clinical management of ailments such as stroke and SIS, thus allowing practitioners to evaluate the effectiveness of various treatment strategies. Since this ratio is calculated based on AGT or AHD, this method may be also suitable for evaluating or predicting the likelihood of SIS or rotator cuff tears.

Based on the correlation between the GHS and the soft tissues injury of shoulder in patients with hemiplegia following a stroke, this ratio may serve as an objective and measurable predictor of early injury. It could be applied to stroke patients to monitor potential worsening over time. Research indicates a significant correlation between the range of motion of external rotation in adolescent overhead athletes and their measured distance (40). This ratio may also be used to predict an athlete’s competitive advantage.

The evaluation of shoulder subluxation in individuals with hemiplegia following a stroke is performed while the arm is in an unsupported, gravity-dependent posture. This position yields reliable results for ultrasound assessment of subluxation (29). To prevent and manage GHS in cases of post-stroke hemiplegia, appropriate positioning is recommended (17). Various techniques aimed at managing GHS in clinical settings have been implemented to support the affected upper limb, including the use of pillows, wheelchair attachments, strapping, and slings (41). The application of ultrasound’s AGT has been utilized to assess changes in GHS resulting from these assistive devices. A novel evaluation approach known as the AGT ratio may facilitate a more efficient cross-sectional analysis of treatment effectiveness, thereby eliminating the need for pre- and post-treatment evaluations.

Multiple investigations indicate that arm positioning significantly influences AGT measurements in both healthy individuals and those with medical conditions (42, 43). Kalra et al. (42) found notable variations in AGT measurements across three distinct postures in both healthy subjects and patients with rotator cuff issues. Similarly, there is a marked alteration in AGT measurements when transitioning from a neutral shoulder position to 60 degrees of abduction (43). Some investigations have also verified the impact of changes in shoulder position on AGT (44, 45). For this study, we employed a neutral posture, allowing the affected arm to hang naturally with the palm oriented towards the trunk. This approach enhances the stability of the upper limb while minimizing uncertainties from upper limb support. Consequently, it is essential to consider the position of the patient’s upper limb when utilizing this ratio.

Although these encouraging findings exist, the present research has various limitations. Firstly, the study employed a small, relatively young convenience sample, consequently, additional research is necessary to evaluate how inter-rater reliability varies with the examiner’s experience in a larger population. Secondly, given the intricate spatial structure of the shoulder joint, it is essential to further investigate GHS in three dimensions. Thirdly, given the limited size of the sample, the r value observed in the correlation analysis is suboptimal. This could be attributed to the size of the sample or the tools used for measurement. In the future, increasing the sample size will be essential for further validating the experimental results mentioned earlier. At last, This study strictly adhered to established inclusion and exclusion criteria, focusing on stroke patients with hemiplegia and GHS, without any artificial intervention in the selection process. However, the distribution of stroke types among the included patients does not correspond with previous reports (46). And there is also a wide time range of disease duration. Further analysis with a larger sample size is required to determine whether multiple factors, such as the types or localizations of stroke, disease duration and so on, is related to GHS.

## 5 Conclusion

This research initially suggests that the bilateral AHD and AGT ratios are used to evaluate the GHS by radiographic and ultrasound techniques demonstrates the reliability and validity. It can provide a comprehensive approach to assessing GHS, enhancing the accuracy and precision of the evaluation. The ratios may also be valuable for predicting dynamic shoulder joint injuries in stroke patients. Furthermore, the findings underscore the importance of early identification and management of GHS in stroke patients, as timely intervention could potentially mitigate the progression of shoulder joint injuries.

## Data Availability

The original contributions presented in the study are included in the article/supplementary material, further inquiries can be directed to the corresponding author.
